# Targeted Therapy with a Novel Superantigen-based Fusion Protein Against Interleukin-13 Receptor α2-overexpressing Tumor Cells: An In-silico Study

**DOI:** 10.30699/IJP.2024.2014231.3200

**Published:** 2024-02-15

**Authors:** Zahra Gholipour, Abbas Ali Imani Fooladi, Kazem Parivar

**Affiliations:** 1 *Department of Biology, Science and Research Branch, Islamic Azad University, Tehran, Iran*; 2 *Applied Microbiology Research Center, Systems Biology and Poisonings Institute, Baqiyatallah University of Medical Sciences, Tehran, Iran*

**Keywords:** Fusion protein, Glioblastoma multiforme, Interleukin-13, Molecular docking, Staphylococcal enterotoxin B.

## Abstract

**Background & Objective:**

Superantigens are bacterial toxins that induce a massive immune response in the host. Superantigen staphylococcal enterotoxin B (SEB) can form a ternary complex with its receptors, MHC class II (MHCII) and TCR, and can be used in tumor-targeting therapy, particularly when cooperating with a specific vector. In this study, SEB was fused to interleukin-13 (IL13), which forms a complex with IL13 receptor α2 (IL13Rα2) overexpressed in glioblastoma multiforme (GBM) cells for therapeutic goals.

**Methods:**

We designed four fusion proteins based on the arrangement of SEB (N- or C-terminal domain) and provided a flexible inter-domain linker (no or yes), resulting in the formation of SEB-IL13, SEB-L-IL13, IL13-SEB, and IL13-L-SEB, respectively. These fusion proteins were then evaluated for their various physicochemical properties and structural characteristics. Bioinformatics tools were employed to predict, refine, and validate the three-dimensional structure of the fusion proteins. In addition, the fusion proteins were docked with IL13Rα2, MHCII, and TCR receptors through the HADDOCK 2.4 server. The candidate fusion protein was subjected to molecular dynamics simulation.

**Results:**

There were differences among the designed fusion proteins. The model with the N-terminal domain of IL13 and containing an inter-domain linker (IL13-L-SEB) was stable and had a long half-life. The docking analysis revealed that the IL13-L-SEB fusion protein had a higher binding affinity to the IL13Rα2, MHCII, and TCR receptors. Finally, using molecular dynamics simulation through iMODS, acceptable results were obtained for the IL13-L-SEB docked complexes.

**Conclusion:**

The results suggest IL13-L-SEB is a promising novel fusion protein for cancer therapeutic application.

## Introduction

Gliomas are the most common primary brain tumors in adults, accounting for around 81% of malignant tumors occurring in the central nervous system (1, 2). The overall survival time of high-grade glioma patients, including glioblastoma multiforme (GBM), is approximately 15 months (2). In GBM cells, certain receptors, including interleukin-13 (IL13) receptor α2 (IL13Rα2), are significantly up-regulated and overexpressed (3-5). IL13Rα2 has an extraordinarily high affinity for IL13 and may regulate IL13 activity by acting as a decoy receptor and dominant-negative regulator of IL13 (6). However, recent studies have suggested that IL13Rα2 may have a signaling function, specifically in cancer cells. The expression of IL13Rα2 is often associated with invasion, metastasis, and advanced stage in tumor cells (7, 8); therefore, this receptor has the potential to be considered for tumor-specific therapeutics (8-10). 

One of the harmful substances generated by *Staphylococcus aureus* bacteria is known as staphylococcal enterotoxin B (SEB), which falls under a category of toxins referred to as staphylococcal enterotoxins. These superantigens can stimulate a large number of T cells and trigger an extensive immune reaction by directly binding to their receptors, MHC class II α-chain (MHCII) and TCR β-chain variable (TCR), without requiring processing by antigen-presenting cells (11, 12). By using a targeted vector, staphylococcal enterotoxins can be delivered specifically to tumor cells, minimizing the side effects associated with non-specific T-cell activation (11).

In the realm of protein engineering, a fusion protein emerges through the amalgamation of two or more distinct domains, constituting what is known as a multi-domain protein (13). The direct fusion of functional domains without a linker may impair the proper stability, folding, expression, or bioactivity of the fusion protein; therefore, the optimal linker peptide might be crucial for its function (14). Inter-domain linkers refer to peptide segments that covalently connect two adjacent domains within a protein (15). The (GGGGS)_3_ peptide is one of the most commonly used flexible linkers, which can provide good solubility, flexibility, and expression, maintain a proper distance between domains, and is appropriate when certain movements are needed for the domains (14, 16, 17). In previous studies, IL13 effectively targeted the IL13 receptor on tumor cells when it was linked directly (IL13-*Pseudomonas *exotoxin A) (9) or via a linker (diphtheria toxin-IL13) to a toxin (10). Similarly, the superantigenic activity of SEB was successfully preserved when fused to a carrier moiety for targeting activity, with (MG7-scFv/SEB) (18) or without (bFGF/SEB) an inter-domain linker (11). 

The three-dimensional (3D) structure plays a crucial role in establishing the biological activity or functionality of a protein (19). Scientists use computational modeling to predict the 3D structure and properties of a protein and even to design or modify new proteins for a desired function (20). A fusion protein with known tertiary structures of composing domains in the RCSB Protein Data Bank (PDB) (21) can be predicted using template-based modeling (i.e., homology modeling) (22). The aim is to explore how the different domain structures can be arranged in 3D to have a low-energy stable arrangement (23, 24). Phyre2 is a suite of modeling tools available on the web that utilizes advanced remote homology detection approaches to construct 3D models and can predict multi-domain proteins (25). 

In this study, to effectively target the IL13Rα2 receptor on GBM cells and finally eliminate the tumor cells, four fusion proteins were designed based on different arrangements of SEB (amino- or carboxy-terminal) and providing an inter-domain linker (no or yes), and they were compared using various bioinformatics tools to find a proper one. The 3D structure of the designed fusion proteins was predicted, refined, and validated. Subsequently, the protein-protein molecular docking analysis of fusion proteins with the receptors was performed, while comparing their binding affinity. Eventually, the stability of ligand-receptor docked complexes was determined by employing molecular dynamics (MD) simulation.

## Material and Methods

**Primary Structure**

**Fig. 1:**
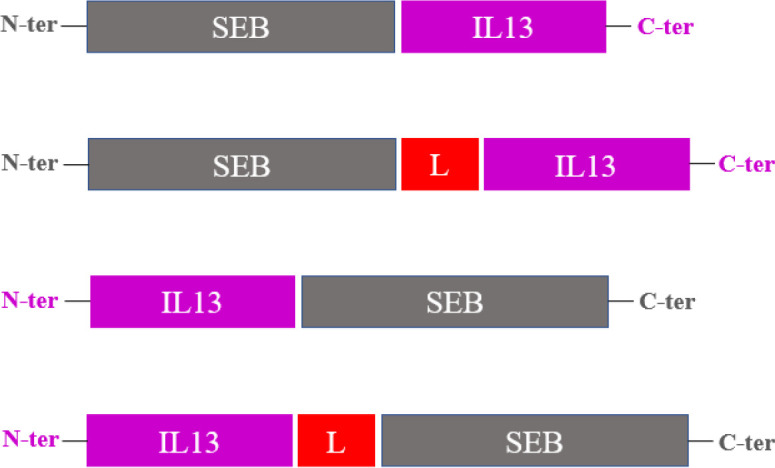
Schematic illustration of the designed SEB-IL13, SEB-L-IL13, IL13-SEB, and IL13-L-SEB fusion proteins, L (linker peptide); GGGGSGGGGSGGGGS.

In the current study, to design a novel fusion protein, SEB (UniprotKB code P01552) was fused either to the N- or C-termini of human IL13 (UniprotKB code P35225), with or without an inter-domain linker peptide (L; GGGGSGGGGSGGGGS). These constructs were termed SEB-IL13, SEB-L-IL13, IL13-SEB, and IL13-L-SEB, respectively (Figure 1). 

**Physicochemical Parameters Prediction**

The physicochemical properties of the designed fusion proteins were predicted from their primary structures (i.e., the full-length amino acid sequences) using the Expay ProtParam server (https://web.expasy.org/protparam/) (26). These properties include the molecular weight (MW), number of amino acids, Theoretical isoelectric point (pI), instability index (II; indicating protein stability in vitro, where values ≤ 40 and > 40 indicate that the protein is stable and unstable, respectively), grand average of hydropathy (GRAVY; indicating the interaction of proteins with water), aliphatic index (AI; related to thermostability), half-life, and extinction coefficient (EC; useful in spectrophotometry experiments).

**Tertiary and Secondary Structures Prediction**

The 3D structures of the four fusion proteins were predicted using Phyre2 (http://www.sbg.bio.ic.ac.uk/phyre2), a web server for homology-based modeling (27). Phyre2 utilizes the Hidden Markov approach to produce alignments of a submitted protein sequence against proteins with published/known structures as templates. The resulting alignments are then used to produce template-based models of the query sequence to predict its 3D structure. Additionally, Phyre2 employs an *ab initio* folding simulation named Poing to simulate regions of a query with no detectable similarities to known structures, such as linker regions in multi-domain proteins. Poing combines a multitude of known structures to generate the final model of the query sequence (25, 28). For modeling with Phyre2, the full-length amino acid sequences of the designed fusion proteins (each fusion protein in a separate session) were submitted, along with choosing the intensive mode. When the modeling process was completed, the results were downloaded in PDB format. The predicted 3D structures, in PDB format, were then subjected to energy minimization/refinement using the GalaxyWEB server (http://galaxy.seoklab.org/cgi-bin/submit.cgi?type=REFINE) to attain minimum energy confirmation and to obtain a thermodynamically stable structure (29). The resulting 3D structure models were visualized using PyMOL software (v1.8.2) (30). Due to the strong coupling between secondary and tertiary structure formation in protein folding (31), information about the secondary structural elements of individual domains of fusion proteins (i.e., IL13 and SEB) was derived from PDB files of energy-minimized 3D structures using PyMOL. 

**Evaluation of 3D Predicted Structures**

The quality of the predicted energy-minimized 3D structures was evaluated using the MolProbity (http://molprobity.biochem.duke.edu/) (32), PROCHECK (https://saves.mbi.ucla.edu/) (33), and ProSA web (https://prosa.services.came.sbg.ac.at/prosa.php) (34) servers. MolProbity analysis is utilized by the critical assessment of the protein structure prediction (CASP) community and evaluates the side-chain conformation quality of the 3D structure. The PROCHECK Ramachandran plot is used to check the stereochemical quality of the backbone structure of proteins. The ProSA-web Z-score measures the energy of proteins of the same size, where a high Z-score and a structure that is relevant to it thermodynamically are inconsistent with each other. 

The entire IL13 and SEB domains of predicted models were aligned versus their respective template structures using PyMOL, and the Cα RMSD was measured. The RMSD calculates the Cα backbone distances (in Å) among aligned structures. 

**Protein-Protein Docking**

The molecular docking online server HADDOCK 2.4 (https://bianca.science.uu.nl/haddock2.4/) (35, 36) was used to investigate the ability of modeled fusion proteins (from the IL13 domain) to bind to IL13Rα2 (fusion protein/IL13Rα2 complex) and the ability of modeled fusion proteins (from the SEB domain) to bind to both MHCII and TCR (MHCII/fusion protein/TCR complex). HADDOCK is an information-driven, flexible docking approach that follows three computational steps: (1) rigid-body energy minimization, (2) semi-flexible refinement in torsion angle space, and (3) final refinement in explicit solvent refinement. For docking, the crystal structures of the IL13/IL13Rα2 (PDB code 3LB6) (6) and MHCII/SEB/TCR (PDB code 4C56) (37) complexes were used. Water molecules and non-bonded ions were removed from the crystal structures, and then the IL13Rα2 as well as MHCII+TCR structures were prepared in separate PDB files using PyMOL. 

To create each complex (i.e., 8 complexes), after submitting the corresponding PDB files, the interface residues (as active residues in HADDOCK) were provided as input. The passive residues (surrounding surface residues) were automatically defined by the software (as default). Finally, the best HADDOCK docking solution for each complex was selected based on the size of the cluster, where the largest cluster has the highest HADDOCK score (computed according to the weighted summation of the van der Waals energy, electrostatic energy, desolvation energy, violation energy, and buried surface area), indicating the strongest binding affinity between the proteins in the complex. Additionally, the binding affinity of IL13 and SEB (crystal structure) to receptors was assessed. The interaction pattern of complexes of the best fusion protein was analyzed with LigPlot^+^ (https://www.ebi.ac.uk/thornton-srv/software/LigPlus/) (38). 

**Molecular Dynamics Simulation**

To assess the stability of protein-protein complexes, a molecular dynamics simulation was conducted. The molecular dynamics study of the docked complexes of the candidate fusion protein (based on the docking score) was performed using the iMODS web server (http://imods.chaconlab.org/). It is an effective, user-friendly, and fast tool for molecular dynamics simulation, which can be utilized effectively to explore the structural dynamics of protein complexes. The stability of the protein is demonstrated in terms of its main-chain deformability plot, eigenvalue, variance, B-factor values, elastic network data, and covariance map (39, 40).

## Results

**Physicochemical Characterization of Fusion Proteins**

According to the physicochemical characterization of the designed fusion proteins, the fusion proteins without the linker (SEB-IL13 and IL13-SEB) comprised 351 amino acids with a molecular weight (MW) of 40.7 kilo Daltons (kDa), whereas those containing the linker (SEB-L-IL13 and IL13-L-SEB) comprised 366 amino acids with a MW of 41.6 kDa. The most frequent amino acid present in the sequence of fusion proteins without or with the linker was Lys (11.4% and 10.9 %, respectively). The total number of positively charged residues (Arg + Lys) and negatively charged residues (Asp + Glu) for all models were 50 and 45, respectively. The isoelectric point (pI) values of the fusion proteins containing SEB as the N-terminal domain were 8.61, and for those containing IL13 as the N-terminal domain, it was 8.60. The instability index of all fusion proteins was smaller than 40. However, the values for proteins without the linker were slightly lower than those containing the linker. Specifically, the instability index was 34.5 and 34.7 for proteins without the linker, and 36.5 and 36.9 for proteins containing the linker, respectively. The GRAVY of SEB-IL13 and IL13-SEB was -0.620, while this value was -0.614 for SEB-L-IL13 and IL13-L-SEB. In this study, the aliphatic index (AI) of fusion proteins without and with the linker was calculated as 73.5 and 70.5, respectively. The estimated half-life (mammalian reticulocytes, in vitro) of fusion proteins with the N-terminal domain of SEB was 1 hour, whereas for the fusion proteins with the N-terminal domain of IL13, the estimated half-life was 30 hours. The extinction coefficients (M^-1^cm^-1^ at 280 nm measured in water) were 44155 for all fusion proteins with absorption of 0.1% (1 g/l) 1.06, assuming all pairs of Cys residues form cystines.

**Structure Modeling of the Fusion Proteins**

**Fig. 2:**
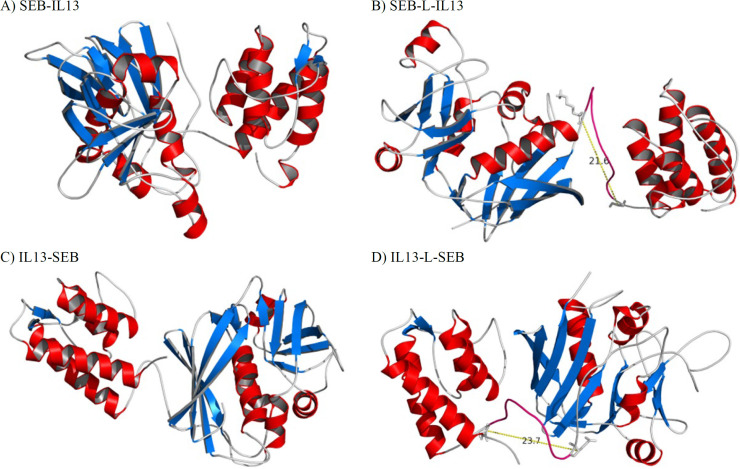
The 3D structure of the final (after energy minimization) (A) SEB-IL13, (B) SEB-L-IL13, (C) IL13-SEB, and (D) IL13-L-SEB fusion proteins predicted by Phyre2. The red color depicts the α-helix, the blue color depicts the ß-strand, and the gray color depicts a random coil. The conformation of the linker is shown in pink. Side chains (as sticks) are displayed for the last residue of the N-terminal domain and the first residue of the C-terminal domain in the models containing the inter-domain linker.

Comparative or homology modeling is a reliable method for predicting tertiary structure based on given amino acid sequences. When the sequences of IL13 and SEB were accessed, Phyre2 homology modeling was used to model four fusion proteins, without (SEB-IL13 and IL13-SEB) or with (SEB-L-IL13 and IL13-L-SEB) an inter-domain linker. The GalaxyWEB server, which can detect unreliable regions and refine loop regions to improve models, was then used to refine each of the models in our study (29). The structures predicted by homology modeling tools rely on the templates used to construct the models. Human IL13 with PDB code 1IK0 (41) and staphylococcal enterotoxin B with PDB code 1D6E (42) were automatically used by Phyre2 as the templates (with a confidence level of 100 %) to create IL13 and SEB structures, respectively, for all models. The 3D structure models of fusion proteins are displayed in Figure 2. In this study, the linker region was modeled in an *ab initio* manner for the IL13-L-SEB and SEB-L-IL13 models. In both models, the linker region was predicted as a random coil. As seen in Figure 2, there was a proper distance between the last residue of SEB (Lys239) and the first residue of IL13 (Gly1) in the SEB-L-IL13 model (distance = 21.6 Å) and between the last IL13 residue (Asn112) and the first SEB residue (Glu1) in the IL13-L-SEB model (23.7 Å). 

The secondary structure plays an important role in characterizing protein structures as it provides a basis for the tertiary structure (31). Given that, we further derived the secondary structure information of individual domains of fusion proteins as well as for those of references (templates) from PDB files of 3D structures (Table 1). In all predicted IL13 structures, α-helices were found to be more frequent, followed by random coils along with very few β-strands. However, in all predicted SEB structures, random coils were found to be more frequent, followed by β-strands along with less frequent α-helices.

**Table 1:** Secondary structural elements of the individual domains of predicted fusion proteins and their respective known structures in PDB

	Fusion Proteins				
Item	SEB-IL13	SEB-L-IL13	IL13-SEB	IL13-L-SEB	References ^1^
Percentage					
IL13					
α	51.8	53.6	58	51.8	56.6
β	5.4	0.00	5.4	5.4	5.3
L	42.8	46.4	36.6	42.8	38.1
SEB					
α	21.3	21.8	22.6	18.2	20.5
β	29.7	30.5	32.2	31.8	34.3
L	49	47.7	45.2	50	45.2

α = α-helix; β = β-strand; L = loop

^1^ The PDB codes 1Ik0 and 1D6E were used as reference.

**Validation of 3D Structure of the Modeled Fusion Proteins**

Validation of the model is crucial in protein structural prediction, as it plays a vital role in designing subsequent experiments and understanding the biological function of the protein. Therefore, the resulting energy-minimized models were validated using various metrics to identify the top tertiary structural models (Table 2). Among the MolProbity metrics, the IL13-L-SEB model was most often considered the best. The Phi/Psi Ramachandran plot statistics were also used to assess the stereochemical quality of structures by quantifying the residues in the allowed zones of the Ramachandran plot. More than 90 % of their residues had proper Psi and Phi torsion angles and were placed in the low-energy areas, indicating good geometry for structures of all models. Indeed, 98.1±0.67 % of amino acids in all models were in both the most favored and additional allowed regions. The ProSA web Z-scores of the energy-minimized models were also calculated. Based on the ProSA web Z-score, it was observed that the IL13-SEB model was slightly better than the SEB-IL13 model (-7.76 vs. -7.74), and SEB-L-IL13 had better quality than the IL13-L-SEB model (-8.85 vs. -8.67). PyMOL was used to align the predicted models with the reference structures (1Ik0 and 1D6E) in entire domains and provide RMSD values (Table 2).

**Table 2:** MolProbity, PROCHECK Ramachandran plot, ProSA web Z-Score statistics, and RMSD value for evaluation of the predicted energy-minimized fusion proteins

	Fusion proteins			
Item	SEB-IL13	SEB-L-IL13	IL13-SEB	IL13-L-SEB
MolProbity				
Clash score	11.41 (65%)	5.85 (91%)	15.98 (46%)	5.15 (93%)
Poor rotamers	1.24	1.23	0.31	0.00
Favored rotamers	96.58	97.85	98.45	99.39
Ramachandran outliers	1.43	0.55	0.86	0.82
Ramachandran favored	94.84	95.88	96.28	96.99
MolProbity score	2.00	1.67	1.96	1.45
PROCHECK Ramachandran plot^1^				
Most favored, %	90.7	93.5	92.3	91.7
Additional allowed, %	6.5	5.2	6.2	6.4
Generously allowed, %	1.2	0.3	0.9	0.6
Disallowed, %	1.6	0.9	0.6	1.2
ProSA web Z-Score	−7.74	−8.85	−7.76	−8.67
RMSD value				
IL13	1.31	1.31	1.32	1.3
SEB	0.38	0.39	0.46	0.34

^1^ Total number of non-glycine and non-proline residues in the structures were 322, 325, 323, and 326 in SEB-IL13, SEB-L-IL13, IL13-SEB, and IL13-L-SEB models, respectively.

**Docking Analysis of Interactions Between Modeled Fusion Proteins and IL13Rα2 or MHCII, and TCR**

As IL13 and SEB proteins have specific binding sites with their respective receptors (IL13Rα2, MHCII, and TCR), the positioning of domains in the designed fusion proteins (as N- or C-terminal domain) would be crucial to enhance the binding activities of fusion proteins with their receptors. The docking of fusion proteins with their receptors (IL13Rα2, MHCII, and TCR) was performed using HADDOCK (Tables 3 and 4). Concerning the fusion protein/IL13Rα2 interactions, the HADDOCK score followed this order: IL13-L-SEB/IL13Rα2 (-179.9±1.9 kcal/mol) > SEB-L-IL13/IL13Rα2 (-162.2±2.8 kcal/mol) > IL13-SEB/IL13Rα2 (-157.7±3.1 kcal/mol) > SEB-IL13/IL13Rα2 (-150.0±9.4 kcal/mol). A detailed analysis showed that van der Waals interaction and surface area were greatest for the IL13-L-SEB/IL13Rα2 complex. It is evident from the HADDOCK analysis that IL13-L-SEB had a slightly higher preference for IL13Rα2 than the other models, which may be useful in selectively targeting IL13Rα2. Interestingly, compared with IL13, the IL13-L-SEB fusion protein showed an increase in IL13Rα2 binding activity. 

For the MHCII/fusion protein/TCR interactions, the HADDOCK score exhibited the following order: MHCII/IL13-L-SEB/TCR (-161.2± 2.2 kcal/mol) > MHCII/IL13-SEB/TCR (-115.9±3.1 kcal/mol) > MHCII/SEB-L-IL13/TCR (-109.2±5.6 kcal/mol) > MHCII/SEB-IL13/TCR (-106.1±5.2 kcal/mol). Similarly, the HADDOCK score results were explained by the greatest van der Waals interaction and surface area for the MHCII/IL13-L-SEB/TCR complex, and therefore, the highest affinity of the IL13-L-SEB model to bind with its receptors. 

**Fig. 3:**
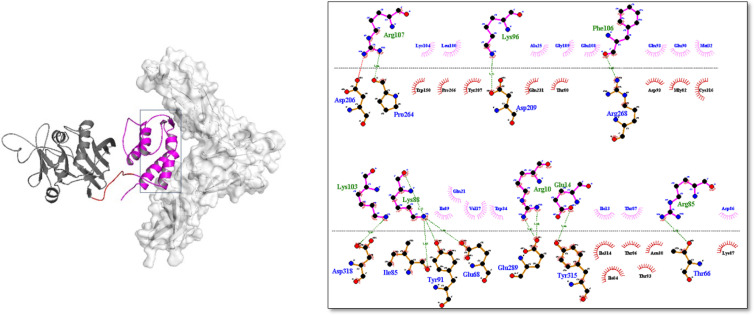
The 3D structure of IL13-L-SEB (IL13, linker, and SEB are depicted in purple, red, and dark gray, respectively) in complex with IL13Rα2 (white). 2D interactions of IL13-L-SEB with IL13Rα2; hydrogen bonds and salt bridge bonds are represented by green and red dash lines, respectively, whereas hydrophobic interactions are indicated by spoked arcs.

**Fig. 4:**
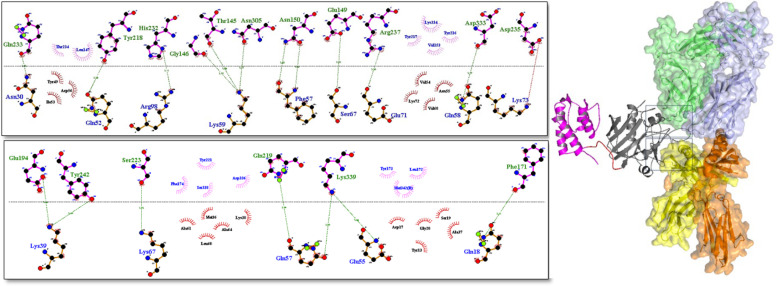
The 3D structure of IL13-L-SEB (IL13, linker, and SEB are depicted in purple, red, and dark gray, respectively) in complex with MHC class II α-chain (yellow) and TCR β-chain variable (green). 2D interactions of IL13-L-SEB with MHCII and TCR; hydrogen bonds and salt bridge bonds are represented by green and red dash lines, respectively, whereas hydrophobic interactions are indicated by spoked arcs.

The results of the best HADDOCK docking pose for IL13-L-SEB/IL13Rα2 and MHCII/IL13-L-SEB/TCR are illustrated in Figures 3 and 4. 

**Table 3:** Top docking models of fusion protein/IL13Rα2 complexes

	Fusion protein/IL13Rα2 complexes				
Item	SEB-IL13/IL13Rα2	SEB-L-IL13/IL13Rα2	IL13-SEB/IL13Rα2	IL13-L-SEB/IL13Rα2	IL13/IL13Rα2
Cluster rank	1	1	1	1	1
HADDOCK score	−150.0 ± 9.4	−162.2 ± 2.8	−157.7 ± 3.1	−179.9 ± 1.9	−173.6 ± 2.8
Cluster size	140	47	67	104	140
RMSD, Å	0.9 ± 0.7	0.3 ± 0.2	2.3 ± 1.3	1.6 ± 1.8	0.4 ± 02
Van der Waals energy, kcal/mol	−51.1 ± 5.9	−58.1 ± 4.4	−54.3 ± 7.2	−66.9 ± 2.1	−64.6 ± 4.6
Electrostatic energy, kcal/mol	−543.5 ± 43.5	−521.9 ± 25.7	−529.2 ± 30.8	−598.6 ± 15.1	−544.0 ± 24.9
Desolvation energy, kcal/mol	4.8 ± 1.3	−2.1 ± 2.0	−0.1 ± 2.2	3.1 ± 4.3	−6.6 ± 2.2
Restraints violation energy, kcal/mol	50.3 ± 26.2	24.2 ± 15.8	25.1 ± 11.0	36.0 ± 6.1	64.2 ± 23.8
Buried surface area, Å	2108.3 ± 95.0	2054.3 ± 41.8	2164.6 ± 116.7	2401.5 ± 121.2	2108.2 ± 25.1
Z-Score	−1.7	−2.1	−2.0	−1.9	−1.6

**Table 4:** Top docking models of MHCII/fusion protein/TCR complexes

	MHCII/fusion protein/TCR complexes				
Item	MHCII/SEB-IL13/TCR	MHCII/SEB-L-IL13/TCR	MHCII/IL13-SEB/TCR	MHCII/IL13-L-SEB/TCR	MHCII/SEB/TCR
Cluster rank	6	5	2	1	1
HADDOCK score	−106.1 ± 5.2	−109.2 ± 5.6	−115.9 ± 3.1	−161.2 ± 2.2	−222.1 ± 1.9
Cluster size	7	11	38	157	200
RMSD, Å	15.7 ± 0.4	0.9 ± 0.6	13.0 ± 1.1	0.7 ± 0.5	1.0 ± 0.6
Van der Waals energy, kcal/mol	−63.5 ± 6.2	−79.7 ± 8.2	−54.0 ± 4.8	−93.6 ± 9.8	−110.8 ± 2.6
Electrostatic energy, kcal/mol	−411.1 ± 23.1	−270.5 ± 37.9	−477.9 ± 23.8	−324.8 ± 44.4	−483.6 ± 27.4
Desolvation energy, kcal/mol	3.5 ± 3.5	−3.5 ± 2.7	5.5 ± 6.4	−14.7 ± 4.7	−16.7 ± 2.1
Restraints violation energy, kcal/mol	361.6 ± 86.7	281.0 ± 17.2	282.6 ± 35.5	121.0 ± 27.8	21.2 ± 7.2
Buried surface area, Å	2240.3 ± 84.8	3064.7 ± 116.3	2305.8 ± 95.1	3302.8 ± 130.1	3414.4 ± 14.2
Z-Score	−2.4	−2.5	−1.7	−1.0	0.0

**Molecular Dynamics Simulation**

**Fig. 5:**
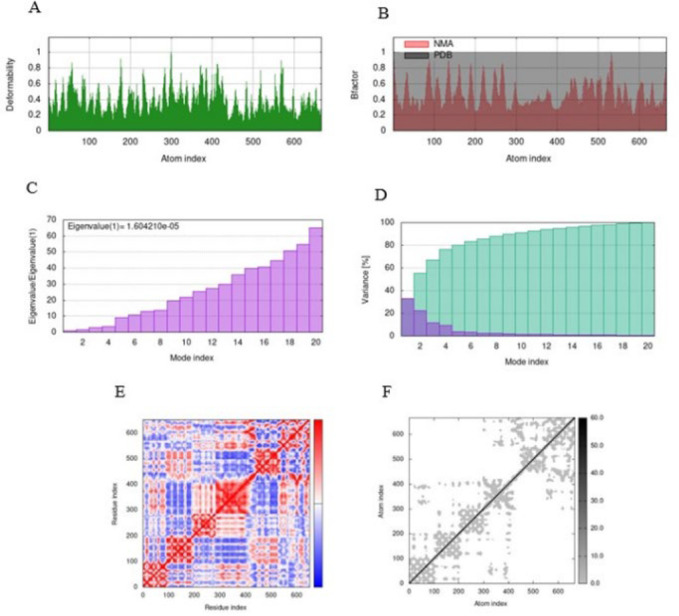
Molecular dynamics simulation of the IL13-L-SEB/IL13Rα2 docked complex. (A) Main chain deformability graph, (B) B-factor analysis, (C) Eigenvalue plot, (D) Variance plot, (E) Covariance matrix, (F) Elastic network model.

**Fig. 6:**
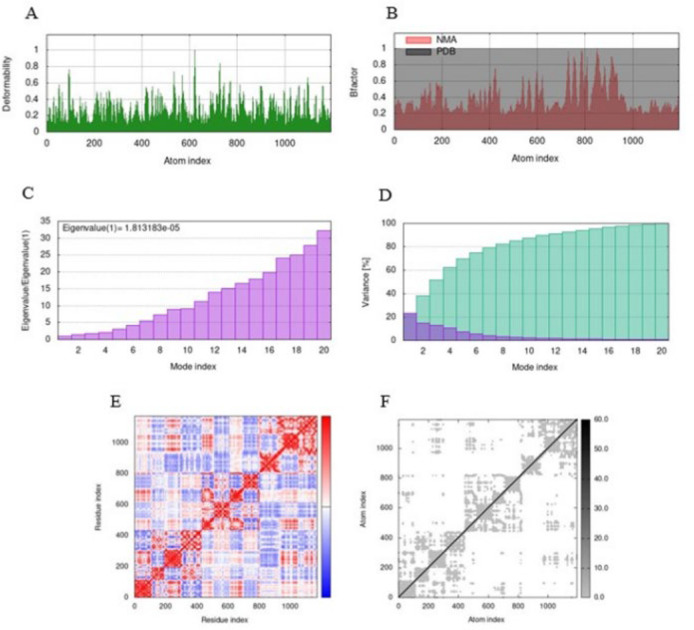
Molecular dynamics simulation of the MHCII/IL13-L-SEB/TCR docked complex. (A) Main chain deformability graph, (B) B-factor analysis, (C) Eigenvalue plot, (D) Variance plot, (E) Covariance matrix, (F) Elastic network model.

After investigating the docking results, it was evident that IL13-L-SEB had a higher binding affinity and was selected for optimization using MD simulation. The MD simulation results of the IL13-L-SEB/IL13Rα2 and MHCII/IL13-L-SEB/TCR docked complexes are depicted in Figures 5 and 6, respectively. The peaks of the graphs corresponding to the regions with deformability in the protein are illustrated using the deformability graphs of the complexes; higher hinges suggest increased flexibility, while lower hinges represent more rigidity in the region (Figures 5A and 6A). Visualization and easy understanding of the comparison between the PDB and NMA fields of the complex can be drawn from the B factor graphs of the complexes, as shown in Figures 5B and 6B. Information about the motion stiffness is provided by the eigenvalue associated with each normal mode. This also provides key insights into the energy needed for deforming structures. Low eigenvalues normally represent easier deformation. The eigenvalues of IL13-L-SEB/IL13Rα2 and MHCII/IL13-L-SEB/TCR were 1.6 × 10^−5^ and 1.8 × 10^−5^, respectively (Figures 5C and 6C). In general, an inverse relation was discovered to exist between the variance and the eigenvalue corresponding to each normal mode. The green bars in the variance graph represent cumulative variance while the purple bars represent individual variance (Figures 5D and 6D). The covariance map of the complexes is depicted in Figures 5E and 6E, showing the relationship between a pair of residues. Red color represents correlated motion, white color represents uncorrelated motion, and blue color represents anti-correlated motion. The findings were additionally confirmed by the elastic network models, which show the pairs of atoms inside the protein linked by springs and the relative stiffness attributed to them. The rigidity of the complexes is indicated by dark gray springs (Figures 5F and 6F) (39, 40).

## Discussion

Targeting the cell surface receptors of tumor cells presents a promising approach to designing drugs against cancer. The overexpression and upregulation of IL13Rα2 in different tumor cells make it an appropriate candidate for developing anticancer drugs (9, 43, 44). SEB, a potent superantigen, can stimulate a significant number of T cells and trigger an extensive immune response. This occurs through its interaction with MHCII and TCR, leading to the formation of a ternary complex comprising TCR/SEB/MHCII specifically on tumor cells (11, 45, 46). In the present study, we designed four fusion proteins comprising IL13 and SEB to specifically target GBM cells. To link the IL13 and SEB domains, a linker consisting of 15 amino acids (GGGGS)3 was employed. Peptide inter-domain linkers refer to specific segments of peptides that form covalent connections between adjacent domains within a protein (15). The flexibility of the linker was found to be crucial in maintaining the bioactivity of fusion proteins (47). 

In previous studies, bioinformatics techniques have been employed to design fusion proteins and enhance their efficacy in targeting cancer cells (48, 49, 50). Similarly, in the present study, bioinformatics approaches were utilized to find a proper fusion protein for therapeutic goals. The Expasy ProtParam tool was employed to assess the physicochemical properties of the fusion proteins. The pI represents the pH of the solution in which the net charge of the surface amino acids of a protein equals zero (26). The results of this investigation revealed that all fusion proteins displayed alkaline pI values. Moreover, the instability index of all fusion proteins was below 40, suggesting that the stability of the designed fusion proteins remained unaltered, and all were deemed stable. The more negative the GRAVY value of a protein, the more hydrophilic the nature of the protein and the greater the possibility of interaction between the protein and water (26). The fusion proteins without the linker were found to be more hydrophilic compared to those containing the linker. The AI of a protein is defined as the relative volume occupied by amino acids with aliphatic side chains (26). The thermal stability of all fusion proteins was high, as indicated by the AI value obtained. It has been demonstrated that the N-terminal residue of a protein has a significant impact on its stability in vivo. Specifically, the N-terminal residue plays a crucial role in the mechanism of ubiquitin-mediated proteolytic degradation (N-end rule) (26). In this study, the amino terminus of fusion proteins with the N-terminal domain of SEB was Glu, and its estimated half-life was 1 h. On the other hand, the amino terminus of fusion proteins with the N-terminal domain of IL13 was Gly, and its estimated half-life was 30 h, suggesting a long half-life.

The Phyre2 web server was utilized to model the tertiary structure of four fusion proteins, which were then refined using the GalaxyWEB server. Likewise, in previous studies (51, 52), the Phyre2 web server has been employed to model proteins with multiple domains. In both IL13-L-SEB and SEB-L-IL13 models, the linker region was predicted as a random coil. It has been shown that the linkers (GGGGS)n have proven to be in coil conformation, irrespective of linker length (53-55). A Previous study showed that a distance of approximately 5 Å is not close enough to form strong polar interactions such as salt bridges or hydrogen bonds between the moieties (54). In the current study, there was a proper distance between the last residue of SEB and the first residue of IL13 in the SEB-L-IL13 model and between the last IL13 residue and the first SEB residue in the IL13-L-SEB model. All predicted models were then evaluated by PROCHECK Ramachandran, MolProbity, and ProSA web Z-score servers. To take side-chain conformations into account, MolProbity analysis on all models was performed. The quality assessment analysis by the MolProbity web server showed that the IL13-L-SEB model obtained a better score in comparison with the other fusion proteins. In PROCHECK Ramachandran, protein models have good quality when more than 90 % of their residues are located in the most favored regions (33). The Z-score evaluates the overall quality of a model and assesses the deviation in the total energy of the structure from the energy distribution obtained from random conformations (34). According to the results reported by the ProSA-web, all the Z-scores of the same-sized structures in the PDB were within a reasonable range after comparison with experimental structures. PyMol was used to align the predicted models and that of the template structures in the entire IL13 and SEB domains. The RMSD values in the entire fusion protein for SEB-IL13, SEB-L-IL13, IL13-SEB, and IL13-L-SEB were 1.69, 1.7, 1.78, and 1.64, respectively. 

Understanding the 3D atomic structure of protein-protein interactions is crucial in the process of designing and developing drugs (35). Molecular docking is one of the most widely used approaches for analyzing protein-protein interactions. In this study, the HADDOCK 2.4 server was used to compare the binding affinity of the four fusion proteins to IL13Rα2, MHCII, and TCR receptors. The results showed that the IL13-L-SEB fusion protein had a higher binding affinity due to more negative scores in both the IL13 and SEB-related complexes. Similarly, in our previous in vitro study, we utilized the cell ELISA method to investigate the binding affinity of the IL13-linker-SEB fusion protein in GBM cells, including U251 (IL13Rα2 positive) and T98G (IL13Rα2 negative). The results demonstrated that the fusion protein exhibited a significantly higher binding affinity in U251 cells compared to T98G cells (44). In the study carried out by Lupardus* et al.*, it was found that the site III interface in the IL13/IL13Rα2 complex is dominated by β1 and β2 strands, with several crucial residues such as Met33 and Lys89 contributing to the interface (6). In the current study, Met32 and Lys88 (based on 112 amino acids of IL13) were found to be involved in hydrophobic and hydrogen interactions with IL13Rα2, respectively. The site III interface of IL13/IL13Rα2 displays more tolerance to point mutations, while the high affinity of the site II interface may compensate for any disruption in site III (6). Some of the important residues in the IL13/IL13Rα2 site II interface include Arg11, Glu15, Lys104, Lys105, Phe107, and Arg108 (6). In the current study, Arg10, Glu14, Lys103, and Phe106 residues were involved in hydrogen interactions, while Arg107 formed a hydrogen bond and a salt bridge bond with the receptor. The residue Lys104 formed a hydrophobic interaction with the corresponding residue of IL13Rα2. In the ternary complex of TCR/SEB/MHCII, SEB binds to the side of MHCII, where the Phe44, Leu45, and Phe47 residues are among the important residues for MHCII binding (37). In our study, these residues were referred to as Phe171, Leu172, and Phe174. Phe171 established a hydrogen bond, while Leu172 and Phe174 appeared in hydrophobic interactions with the corresponding residues of MHCII. Regarding the interface between SEB and TCR in the ternary complex of MHCII/SEB/TCR, SEB engages TCR through its TCR-binding cleft, a shallow groove located between its N-terminal β-barrel and the α2 helix (37). Rödström* et al.* found several crucial interface residues responsible for the binding of SEB to TCR, which include Asn23, Val26, Tyr90, and Tyr91 (37). In our study, Asn150 and Tyr218 established hydrogen bonds with the receptor. On the other hand, Val153 and Tyr217 appeared in hydrophobic interactions with the respective residues of the receptor. 

In our previous study, we evaluated the cytotoxic effects of the IL13-linker-SEB fusion protein on GBM cells. The findings indicated that the fusion protein successfully targeted IL13Rα2 on U251 (IL13Rα2 positive) cells and maintained the activity of SEB (44). In the present study, the iMODS server was used to evaluate the stability of two complexes of the IL13-L-SEB fusion protein. The iMODS server can evaluate complex motion and deformability and provide valuable insights into the stability, structural dynamics, and functional properties of protein complexes. Additionally, it enhances our understanding of the biological functions of the complexes, thereby promoting the advancement of therapeutic approaches (39, 40). All the iMODS analyses suggested that the IL13-L-SEB fusion protein had enough stability to be used for therapeutic goals and further evaluation.

## Conclusion

In this study, our goal was to identify a promising therapeutic fusion protein that combines IL13 and SEB domains to target IL13Rα2, MHCII, and TCR receptors using computational methods. The fusion protein, named IL13-L-SEB, with IL13's N-terminal domain and an inter-domain linker, exhibited stability and a strong inclination to interact with its receptors. The MD simulation yielded satisfactory results for the IL13-L-SEB docked complexes. Our computational findings indicate that IL13-L-SEB holds promise as a potential anti-cancer agent against GBM.
